# Association Between Metabolic Health and Bone Mineral Density Using CT in Hepatocellular Carcinoma Patients Under 65: A Retrospective Chart Review

**DOI:** 10.7759/cureus.70835

**Published:** 2024-10-04

**Authors:** Kian A Huang, Haris K Choudhary, Katherine G Quesada-Tibbetts, Neelesh Prakash

**Affiliations:** 1 Radiology, USF Health Morsani College of Medicine, Tampa, USA

**Keywords:** bone mineral density, diabetes, dyslipidemia, hepatocellular carcinoma, osteoporosis

## Abstract

Introduction: Metabolic conditions such as diabetes, and dyslipidemia are prevalent in the United States (US), serving as potential risk factors for hepatocellular carcinoma (HCC). This study aimed to examine the association between various metabolic markers and Hounsfield Units (HU) from L1 vertebral CT scans as indicators of bone mineral density (BMD) in HCC patients under age 65.

Methods: A cross-sectional analysis was conducted on HCC patients under 65. Correlational and regression analyses were used to assess the association of metabolic markers and other health variables with HU scores.

Results: Race and age were significantly associated with HU scores in multivariate analyses, indicating these factors play a crucial role in bone health among HCC patients. Race showed a positive association, and age showed a negative association with HU scores. Fasting blood glucose had a significant negative correlation with BMD, but this relationship was not significant in univariate regression analysis. No significant correlations were found between HU scores and triglycerides, cholesterol, low-density lipoprotein (LDL), high-density lipoprotein (HDL), cholesterol/HDL ratio, LDL/HDL ratio, and hemoglobin A1C (HbA1c) levels.

Conclusion: Traditional metabolic markers may not be strong predictors of osteoporosis in this specific population. Further research with larger, more diverse populations and longitudinal data is necessary to understand better the factors contributing to BMD variations in HCC patients.

## Introduction

Metabolic conditions such as diabetes, and dyslipidemia are prevalent in the United States (US). It is estimated that 11.6% of Americans in 2021 had diabetes and more than one-third of adults in the US have dyslipidemia [1,2]. The metabolic consequences of these disorders have complex and multifaceted impacts on bone health, specifically bone mineral density (BMD). Diabetes is correlated with a significant increase in the risk of bone fracture [3-9]. However, the mechanism for the increased risk of fracture is paradoxical: type 1 diabetes appears to be associated with a decrease in BMD due to a decrease in insulin and amylin and their osteoanabolic effects whereas type 2 diabetes is associated with hyperinsulinemia and an increase in BMD but of lower quality [3-8]. The relationship between dyslipidemia and BMD is mixed. Studies in hyperlipidemic and hypercholesterolemic mice demonstrate lower average BMD in the femur and vertebrae, possibly due to an attenuated response in parathyroid hormone-related bone anabolism [10-13]. In postmenopausal women and older adults, higher high-density lipoprotein (HDL) has been shown to have a positive relationship with higher. Higher total cholesterol and low-density lipoprotein (LDL) have been shown to have both no effect on BMD and negative effects on BMD [13-16].

Diabetes is a risk factor for hepatocellular carcinoma (HCC) and is linked to a two- to three-fold increase in the risk of developing HCC [17-20]. The link between hyperlipidemia and the development of HCC is unclear. Hyperlipidemia is associated with a greater risk of developing HCC in patients without cirrhosis [21-23]. However, some conflicting research has found hyperlipidemia to confer protective effects against developing HCC [22]. These relationships are critical, as HCC is one of the leading causes of cancer-related deaths in the US.

Moreover, HCC patients are particularly vulnerable to osteoporosis due to their unique metabolic profile, including coexisting conditions like diabetes and dyslipidemia. The risk of osteoporosis or fractures is notably higher in patients with liver disease than in the general population. Additionally, liver cirrhosis, a common precursor to HCC, is closely associated with bone mass loss, which becomes more pronounced as liver function deteriorates due to lower muscle mass and frailty, negatively impacting prognosis [24]. Importantly, BMD serves as both an indicator of bone health and a marker of nutritional status, a crucial factor that significantly influences the prognosis and outcomes of HCC. Research has shown that low BMD often precedes the onset of sarcopenia and other musculoskeletal imbalances, which are recognized as important prognostic factors in HCC. In patients with HCC, low BMD is an independent prognostic factor, predicting poorer outcomes and reduced survival, particularly post-liver transplantation [25]. This establishes a bidirectional relationship between BMD and HCC: low BMD may contribute to the onset of HCC by reflecting compromised nutritional status and overall systemic health, exacerbating liver disease, while in those already affected by HCC, it serves as a marker of worse prognosis, especially in post-transplant survival. Given the high prevalence of metabolic disorders in the US and their potential impact on bone health, it is crucial to understand how these disorders specifically affect BMD in HCC patients. The progression from cirrhosis to HCC exacerbates bone loss, further heightening the risk of osteoporosis and its complications in these patients. Dual-energy X-ray absorptiometry (DEXA) scans, the gold standard for assessing BMD, are typically recommended only for those 65 and older [26]. However, considering the potential for earlier onset of low BMD in HCC patients due to their metabolic predispositions and liver dysfunction, there is a strong justification for extending DEXA screening to younger HCC patients under 65. Addressing this gap could lead to earlier detection and management of osteoporosis in this at-risk group, ultimately improving outcomes.

This study uses L1 CT scans as an alternative indicator of low BMD in HCC patients under 65 to analyze the prevalence of low BMD in this population and compare metabolic risk factors such as hyperglycemia, dyslipidemia, serum HDL, serum LDL, and HbA1c. Hounsfield Units (HU) from CT scans are a reliable predictor of T-scores from DEXA scans and can be used to predict osteoporosis risk in patients [27,28]. Researchers have shown that the CT attenuation threshold of an L1 vertebra of less than 160 HU has a 90% sensitivity to osteoporosis, as evaluated with DEXA [26]. Given the prevalence of metabolic disorders in HCC patients, putting them at risk for lower BMD and BMD-related bone diseases such as osteoporosis, this population may benefit from regular DEXA screenings to detect low BMD early and provide effective treatment before further damage ensues. We hypothesize that patients under 65 with HCC will exhibit a higher prevalence of low BMD due to the metabolic effects of diabetes and dyslipidemia.

## Materials and methods

This retrospective cohort study aimed to investigate the impact of various metabolic markers on BMD in young patients under 65 years old (n=170) with HCC. The markers tested were fasting blood glucose, serum triglycerides, serum cholesterol, LDL, HDL, cholesterol/HDL ratio, LDL/HDL ratio, and hemoglobin A1C (HbA1c) levels. Covariates measured included sex, race, ethnicity, Charlson comorbidity index (CCMI), body mass index (BMI), as well as, serum vitamin D, potassium, calcium, albuminin, and ferritin levels. BMD was collected using HU scores measured from the CT of the lumbar spine, specifically of the L1 vertebrae. All data was extracted from the earliest comprehensive metabolic panel (CMP), lipid panel, and lumbar spine CT scan from the electronic health record between January 2019 and January 2021 at Tampa General Hospital (TGH).

The inclusion criteria encompassed patients diagnosed with HCC who underwent a CT scan of their spine, including an assessment of the T1 vertebra, from 2019-2021. Patients missing a CMP or lipid panel were excluded from the analysis to ensure data completeness and reliability.

Descriptives, correlations, and multivariate and univariate regressions were performed with a significance level set at p < 0.05, and all analyses were conducted in SPSS version 29.0 (IBM, New York, US). Descriptives and a Spearman's rho correlation were calculated for every variable within the patient sample. Variables demonstrating a significant correlation with the HU score were then processed with a univariate regression analysis. Variables with a significant association with HU score then underwent a multivariate regression analysis, controlling for the aforementioned covariates known to potentially influence BMD including sex, race, ethnicity, CCMI, and BMI.

## Results

As shown in Table 1, descriptive statistics were calculated for the HU scores of the 170 patients included in the study, with a mean ± standard deviation (SD) of 142.2 ± 44.28. 126 (74.1%) patients within the sample had an HU score of less than 160, which, according to previous literature, is a likely indicator of osteoporosis [26]. The average age ± SD was 58.29 years old ± 6.42, the average BMI ± SD was 28.39 ± 7.30, and the average CCMI ± SD was 6.50 ± 2.20.

**Table 1:** Descriptive statistics of the patient sample Descriptive statistics of the patient sample, including mean, standard deviation, and sample size for variables such as HU score, age, BMI, CCMI, and lab values (e.g., Vitamin D, potassium) HU: Hounsfield unit; BMI: Body mas index; CCMI: Charlson comorbidity index; LDL: Low-density lipoprotein; HDL: High-density lipoprotein; HbA1c: Hemoglobin A1C

	Mean	Standard Deviation	N
HU Score	142.22	44.28	170
Age	58.29	6.42	170
BMI	28.39	7.3	170
CCMI	6.5	2.2	170
Vitamin D	18.48	12.28	32
Potassium	4.23	0.55	170
Calcium	9.05	0.84	170
Albumin	3.29	0.78	170
Ferritin	494.1	851.7	100
Fasting Glucose	128.07	59.73	170
Triglyceride	129.24	94.57	79
Cholesterol	146.86	49.06	88
LDL Cholesterol	84.53	39.65	77
HDL Cholesterol	42.08	19.13	77
Cholesterol/HDL Ratio	4.36	3.37	77
LDL/HDL Ratio	2.76	3.64	76
HbA1c	5.9	1.68	116

For binary characteristics, as reflected in Table 2, 42 (24.7%) patients were female, 36 (21.2%) patients were Hispanic, 27 (15.9%) patients were non-white, and 118 (69.4%) patients had no diagnosis of any type of diabetes.

**Table 2:** Binary descriptive characteristics of patient sample Binary descriptive characteristics (e.g., sex, ethnicity, race, and diabetes status) calculated for the patient sample (n = 170)

Characteristics	Category	Number (Percent)
Sex	Male	128 (75.3)
Sex	Female	42 (24.7)
Ethnicity	Hispanic	36 (21.2)
Ethnicity	Non-Hispanic	134 (78.8)
Race	White	142 (83.5)
Race	Non-white	27 (15.9)
Diabetes	No	118 (69.4)
Diabetes	Yes	52 (30.6)

To compare HU scores with our categorical variables, the Mann-Whitney U Test was employed, revealing that HU scores were significantly different between racial groups. As seen in Table 3, Non-white participants had significantly higher HU scores, with a median of 155.57 (IQR 66.78) compared to a median of 134.37 (IQR 48.34) among white participants (p < 0.001). Although 52 (30.6%) patients within the sample were diagnosed with any form of diabetes, diabetic status did not have a significant impact on HU scores (p = 0.071). Furthermore, although not statistically significant, males (136.42, IQR 45.91) tended to have a lower HU score than females (153.85, IQR 66.92), and non-Hispanics (136.70, IQR 36.09) in the sample had a lower HU score than Hispanics (143.36, IQR 66.91).

**Table 3:** Relationship between categorical predictors and HU scores Results of Mann-Whitney U tests examining differences in HU score by categorical variables (e.g., sex, ethnicity, race, and diabetes status). P-values indicate the statistical significance of differences. The p < 0.05 is considered significant and significant p-values marked with *. HU: Hounsfield unit

Variable	Category	HU Score Median (Interquartile Range)	P-Value
Sex	Male	136.42 (45.91)	0.7
Sex	Female	153.85 (66.92)	0.7
Ethnicity	Hispanic	143.36 (66.91)	0.192
Ethnicity	Non-Hispanic	136.70 (36.09)	0.192
Race	White	134.37 (48.34)	<0.001*
Race	Non-white	155.57 (66.78)	<0.001*
Diabetes	No	138.93 (57.25)	0.071
Diabetes	Yes	128.60 (49.00)	0.071

In Table 4, spearman's rho correlation was utilized to evaluate the potential relationships between continuous variables and HU scores. Age, comprehensive metabolic panel (CMP) potassium, and fasting blood glucose were significantly correlated with HU scores. The analysis demonstrated significant negative correlations between age and HU scores (rho = -0.215, 95% CI -0.358 to -0.062, p = 0.005), CMP potassium levels and HU scores (rho = -0.260, 95% CI -0.399 to -0.109, p = 0.001), and fasting glucose levels and HU scores (rho = -0.160, 95% CI -0.307 to -0.005, p = 0.038). No significant correlations were found between HU scores and serum triglycerides, serum cholesterol, LDL, HDL, cholesterol/HDL ratio, LDL/HDL ratio, and HbA1c levels.

**Table 4:** Correlations between metabolic health markers and HU scores Spearman’s correlation coefficients between continuous variables and HU scores. The p < 0.05 is considered significant and significant p-values marked with *. BMI: Body mass index; CCMI: Charlson comorbidity index; LDL: Low-density lipoprotein; HDL: High-density lipoprotein; HU: Hounsfield unit; HbA1c: Hemoglobin A1C

Variable	Correlation Coefficient	95% Confidence Interval	P-Value
Age	-0.215	(-0.358,-0.062)	0.005*
BMI	-0.033	(-0.187,0.122)	0.668
CCMI	-0.141	(-0.289,0.014)	0.067
Vitamin D	-0.077	(-0.424,0.289)	0.675
Potassium	-0.26	(-0.399,-0.109)	0.001*
Calcium	0.012	(-0.143,0.167)	0.877
Albumin	-0.118	(-0.268,0.038)	0.127
Ferritin	0.049	(-0.155,0.248)	0.631
Fasting Glucose	-0.16	(-0.307,-0.005)	0.038*
Triglyceride	-0.094	(-0.315,0.136)	0.408
Cholesterol	0.114	(-0.104,0.321)	0.292
LDL	0.106	(-0.127,0.328)	0.359
HDL	0.016	(-0.215,0.245)	0.891
Cholesterol/HDL Ratio	-0.04	(-0.268,0.192)	0.73
LDL/HDL Ratio	0.044	(-0.19,0.273)	0.704
HbA1c	0.002	(-0.186,0.189)	0.984

As illustrated in Table 5, further analyses were performed using univariate and multivariate linear regression models to identify predictors of HU scores. In the univariate analysis, fasting glucose was not a significant predictor (β = -0.025, 95% CI -0.138 to 0.088, p = 0.664), whereas race, age, and CMP potassium remained significant predictors. A multivariate linear regression model was then constructed, adjusting for race, age, CMP potassium, and fasting glucose. This model indicated that CMP potassium and fasting glucose were insignificant predictors of HU scores. However, age (β = -1.22, 95% CI -2.24 to -0.210, p = 0.018) and race (β = 32.26, 95% CI 14.98 to 49.54, p < 0.001) were significant predictors, with older age being associated with lower HU scores and non-white race being associated with higher HU scores. The analysis also showed that several continuous predictors did not significantly correlate with HU scores. These included triglyceride levels (rho = -0.094, 95% CI -0.315 to 0.136, p = 0.408), cholesterol levels (rho = 0.114, 95% CI -0.104 to 0.321, p = 0.292), LDL levels (rho = 0.106, 95% CI -0.127 to 0.328, p = 0.359), HDL levels (rho = 0.016, 95% CI -0.215 to 0.245, p = 0.891), cholesterol/HDL ratio (rho = -0.040, 95% CI -0.268 to 0.192, p = 0.730), LDL/HDL ratio (rho = 0.044, 95% CI -0.19 to 0.273, p = 0.704), and A1C levels (rho = 0.002, 95% CI -0.186 to 0.189, p = 0.984).

**Table 5:** Linear regression predicting HU scores Linear regression models predicting HU score. Univariate and multivariate regression coefficients (with 95% confidence intervals) are reported. Significant predictors include race, CCMI, and potassium levels. The p < 0.05 is considered significant and significant p-values marked with *. HU: Hounsfield unit; CCMI: Charlson comorbidity index

	Univariate		Multivariate	
Variable	Estimate (95% Confidence Interval)	P-Value	Estimate (95% Confidence Interval)	P-Value
Race	38.63 (21.49, 55.78)	< .001*	32.26 (14.98, 49.54)	< .001*
Age	-1.72 (-2.73, -0.698)	0.001*	-1.22 (-2.24, -0.210)	0.018*
Potassium	-16.61 (-28.66, -4.56)	0.007*	-10.42 (-22.20, -1.36)	0.082
Fasting Glucose	-0.025 (-0.138, .088)	0.664	-0.020 (-0.086, 0.126)	0.708

## Discussion

The findings of this study underscore the complex relationship between metabolic health and BMD in patients with HCC under 65 years of age. Results demonstrate that non-white race is associated with higher HU scores, indicative of higher BMD, compared to white participants. This finding supports the consensus that non-whites have higher BMD than whites specifically at the lumbar vertebrae. Previous literature has reported that non-white races like non-Hispanic black individuals have a 6% greater lumbar spine BMD than white populations [29]. Age was negatively correlated with HU scores, indicating that younger HCC patients tend to have higher BMD. This aligns with existing knowledge that BMD decreases with age due to various physiological changes, including hormonal changes and increased bone turnover rate driven by reduced bone formation, and increased bone resorption [30]. Additionally, this study identified a significant negative correlation between CMP potassium levels and HU scores, which warrants further investigation to elucidate the underlying mechanisms linking potassium metabolism and bone health in HCC patients.

Interestingly, fasting glucose levels were negatively correlated with HU scores in the initial correlation analysis, but this association did not hold in the univariate regression analysis. This suggests that while there may be a relationship between glucose metabolism and BMD, it is likely influenced by other factors not accounted for in the univariate model. The multivariate regression analysis further confirmed that age and race are significant predictors of HU scores, even after adjusting for CMP potassium and fasting glucose. These findings highlight the importance of considering multiple metabolic factors and demographic variables when assessing bone health in HCC patients. HU scores from CT scans as a surrogate for BMD assessment are particularly relevant for this population, given the common use of CT imaging in HCC management and the need for early identification of osteoporosis or osteopenia in younger patients.

Despite thorough investigation, the analysis showed that several continuous predictors did not significantly correlate with HU scores. These included triglyceride levels, cholesterol levels, LDL levels, HDL levels, cholesterol/HDL ratio, LDL/HDL ratio, diabetic status, and HbA1c levels. There are a few potential reasons for these non-significant findings. First, the lack of significant correlations might be attributed to the relatively young age of the study population, which was restricted to HCC patients under 65 years old. This age group might not exhibit the same metabolic changes or bone density variations typically seen in older populations, where such predictors might play a more prominent role. Second, the specific characteristics and heterogeneity of the HCC population could contribute to these findings. The disease and its treatment regimens might influence metabolic markers and BMD differently compared to the general population or other patient groups. Lastly, the sample size and statistical power of this study might not have been sufficient to detect subtle associations. While the sample size was reasonable, larger studies with more diverse populations might be necessary to uncover significant relationships between these metabolic markers and HU scores. These considerations highlight the complexity of factors influencing BMD in HCC patients and underscore the need for further research to elucidate these relationships more clearly.

One of the primary strengths of this study is its focus on a specific and understudied population (HCC patients under the age of 65). This focus allowed us to explore BMD issues in a group that is often not the target of osteoporosis screening guidelines. This study also supported the use of HU from CT scans as a surrogate marker for BMD as a practical approach, given the accessibility and routine use of CT imaging in clinical practice. Our comprehensive dataset, including various metabolic markers and demographic variables, enabled a detailed analysis of potential predictors of low BMD. This study also identified significant relationships between HU scores and race as well as HU scores and age, corroborating with the existing literature on BMD and supporting the idea that these associations may also extend into the population of young HCC patients. However, there are also notable limitations to this study. Being a retrospective chart review, the study is subject to inherent biases such as selection bias and information bias. The reliance on existing medical records may result in incomplete or inconsistent data collection.

Additionally, the study's cross-sectional design limits the ability to infer causality between metabolic markers and BMD. The relatively small sample size and focus on a single institution may limit the generalizability of the findings to broader populations. Finally, the lack of longitudinal data prevents assessing changes in BMD over time or the long-term impact of metabolic conditions on bone health in HCC patients.

## Conclusions

In this retrospective chart review study, we investigated the relationship between various metabolic markers and HU scores as a surrogate for BMD in HCC patients under age 65. The findings indicate that diabetes status did not significantly impact HU scores, and several continuous predictors, including triglyceride, cholesterol, LDL, HDL, cholesterol/HDL ratio, LDL/HDL ratio, and HbA1c levels, showed no significant correlation with HU scores. These results highlight the complexity of factors influencing bone health in HCC patients and suggest that traditional metabolic markers may not be strong predictors of osteoporosis in this specific population. The study's strengths include its focus on an understudied population, substantiating the literature regarding the impact of age and race on BMD and extending it to young HCC patients, and supporting the practical use of HU scores from CT scans to measure BMD. However, limitations such as selection bias, small sample size, and the cross-sectional design must be considered when interpreting the results. Further research with larger, more diverse populations and longitudinal data is necessary to better understand the factors contributing to BMD variations in HCC patients.
